# Omega-3 Polyunsaturated Fatty Acids for the Treatment of IgA Nephropathy

**DOI:** 10.3390/jcm6070070

**Published:** 2017-07-19

**Authors:** Junichi Hirahashi

**Affiliations:** Apheresis and Dialysis Center, Keio Univerisity School of Medicine, Tokyo 1608582, Japan; jhira@keio.jp

**Keywords:** ω-3 PUFAs, EPA, DHA, IgA nephropathy, aspirin

## Abstract

IgA nephropathy is a common disease that causes end-stage renal failure and requires renal replacement therapy. The main purpose of therapeutic intervention in this disease is not limited to improvement of prognosis and prevention of transition to end-stage renal failure, but also prevention of the occurrence of cardiovascular lesions, which increases risk in patients with chronic kidney disease. Steroids and immunosuppressants have been widely used as remission induction therapies; however, the balance between their therapeutic benefits and detrimental side-effects are controversial. In this regard, it is critical to identify alternative therapies which would provide holistic life-long benefits. Currently, the potential of ω-3 fatty acids as anti-inflammatory and inflammation-convergent drugs—especially the remarkable progress of the multifunctional ω-3 polyunsaturated fatty acids (PUFAs)—has garnered attention. In this section, we outline the background and current status of ω-3 PUFA-based treatment in IgA nephropathy.

## 1. Introduction

IgA nephropathy (IgAN) is the major cause of primary glomerulonephritis, and approximately 1% of the population suffers from this disease undiagnosed. Approximately 150,000 cases of IgAN are reported in the United States, 4000 new cases are diagnosed annually, and 25% patients with IgAN shift to end-stage renal failure within 25 years of diagnosis [1]. IgAN accounts for 50% cases of glomerulonephritis in Japan, and it is a major cause of end-stage renal failure in the young. In many cases, immunosuppressive agents, including steroids, are used for remission induction therapy.

## 2. Significance of Therapeutic Intervention in IgAN

Severe proteinuria, impaired renal function, and hypertension are predictors of progression to end-stage renal failure in patients with IgAN [2,3]. On the contrary, patients with IgAN associated with minimal urine findings and normal range of renal function show good long-term prognosis [4], and such patients do not require immunosuppressive therapy. Mortality in patients with IgAN was twice the expected rate prior to intervention with renal replacement therapy, and it is the most common cause of death due to cardiovascular disease, accounting for 45% of all IgAN-related deaths [5]. The main purpose of therapeutic intervention in this disease is not only to improve prognosis and hinder the transition to end-stage renal failure, but also to prevent the prevalence of cardiovascular lesions, the risk rate of which increases in patients with chronic kidney disease (CKD). Immunosuppressive therapy—including use of steroids—has been widely used for treating autoimmune diseases. In particular, remission induction was achieved using a combination of steroid pulse and tonsillectomy during the treatment of IgAN in Japan [6,7]. However, the adverse side-effects of these drugs pose problems; especially, physicians must consider the effect of these drugs on arteriosclerotic lesions, thrombosis, infection, and diabetes prior to administration to the elderly. Recent STOP-IgAN studies [8] reported that “immunosuppressive therapy for IgAN does not improve prognosis but rather adverse events become a problem”. In connection with the remarkable advances in lipid mediator research in recent years, we outline the treatment of IgAN with fish oil rich in ω-3 polyunsaturated fatty acids (PUFAs) in this study.

## 3. History of Treatment of IgAN with ω-3 PUFAs

Consumption of omega-3 PUFAs—which are abundant in fish oil and possess anti-inflammatory effects—is useful for preventing and treating organ-specific inflammation and systemic inflammatory diseases. Several fundamental research and clinical trials in Japan and abroad investigated whether fish oil rich in docosahexaenoic acid (DHA) or eicosapentaenoic acid (EPA) (ω-3 PUFAs), is effective for the prevention and treatment of IgAN. Studies in a murine model with clinical symptoms of early IgAN induced by vomitoxin—a mold toxin of cereals—showed that ω-3 PUFAs suppress IL-6-induced abnormal IgA production and deposition of IgA immune complexes in the mesangial region [9,10]. Several reports suggest that omega-3 PUFAs inhibit the progression of IgAN in actual clinical practice (Table 1). Hamazaki et al. reported for the first time that renal function improved upon ingestion of 1.6 g of EPA and 1.0 g of DHA per day in 20 patients with IgAN in a randomized trial [11]. Alexopoulos et al. [12] demonstrated that a “very low dose” of omega-3 PUFAs is effective in slowing renal progression in high-risk patients with IgAN [13]. A Polish study reported that ingestion of 0.54 g EPA and 0.81 g DHA per day for 12 months improved renal function and reduced urinary protein content of patients with IgAN. A randomized, double-blinded, maximum-scale clinical trial by the Mayo Nephrology Collaborative Group demonstrated the inhibitory effect of ω-3 PUFAs on the progression of renal dysfunction in 106 high-risk group patients with IgAN and proteinuria (2.8 ± 2.5 g/day). During the 2-year-long treatment with 1.8 g EPA and 1.2 g DHA per day, the proportion of serum creatinine increased by 50% or more was 33%, whereas in the control group (olive oil administered group) the increase was 6% [14]. Renal disorder progression was remarkably suppressed with the combined usage of DHA and EPA, especially in the high-risk group that was characterized by nephrotic level proteinuria, high blood pressure, and decreased renal function. A 6.4-year follow-up [15] revealed a decrease in glomerular filtration rate (GFR) decline rate and significant reduction in the transition to end-stage renal failure (absolute risk reduction of 29%). However, it should be noted that there was no difference in the proteinuria-ameliorating effect between the two groups. Similar improvement was observed upon treatment with low-dose (EPA 1.88 g + DHA 1.47 g/day) and high-dose (EPA 3.76 g + DHA 2.94 g/day) EPA and DHA [16]. These observations highlighted the efficacy of administration of ω-3 PUFAs in early stages of IgAN. Ferraro and colleagues [17] reported a reduction of proteinuria by 72.9% (1.31 ± 1.20) upon administering ω-3 PUFAs for 15 months to 15 patients with IgAN who had been treated with inhibitors of the renin–angiotensin system. However, no decrease in proteinuria was observed in the control group. In addition, a significant decrease in hematuria was observed, which, however, remained unchanged in the control group. Similar results were reported by Japanese researchers who used purified EPA [18]. These findings indicated that ω-3 PUFAs could possibly improve IgAN-associated proteinuria, independent of the renin–angiotensin system. Considering that the prophylactic measures for cardiovascular diseases [19] determine the prognosis of renal diseases and CKD, it is expected that the efficacy of ω-3 PUFAs administration in the early stages of CKD caused by IgAN will be high. Contrary to the above results, certain studies showed that ω-3 PUFAs are not effective for treating human IgAN. In a prospective study in Australia [20], 37 individuals who were evaluated to have IgAN by renal biopsy were prescribed 10 g/day EPA for 2 years. However, EPA did not change the course of IgAN compared to that of the untreated control. In a six-month prospective, randomized, double-blind study conducted by Pettersson et al. [21], 32 Swedish adults with IgAN were treated with fish oil containing high dose of ω-3 PUFAs (6 g/day; EPA 55%, DHA 30%) and the control group was treated with 6 g corn oil per day. Surprisingly, the fish oil-treated group showed no clinical benefit over the control group after 6 months of treatment. In addition, in a randomized double-blind study conducted by Hogg et al. [22], 4 g ω-3 PUFAs (1.88 g EPA + 1.48 g DHA) or placebo was added to prednisone and administered to 96 individuals with IgAN for two years. Comparison of the 3-PUFA-treated group with the placebo group showed that neither slowed the progression of renal failure. As mentioned above, clinical trials with omega-3 unsaturated fatty acid for treating IgA are time-consuming, and may therefore produce conflicting results. Therefore, omega-3 fatty acids have not yet found aggressive clinical application.

## 4. Anti-Inflammatory Actions of ω-3 PUFAs

**Figure 1:**
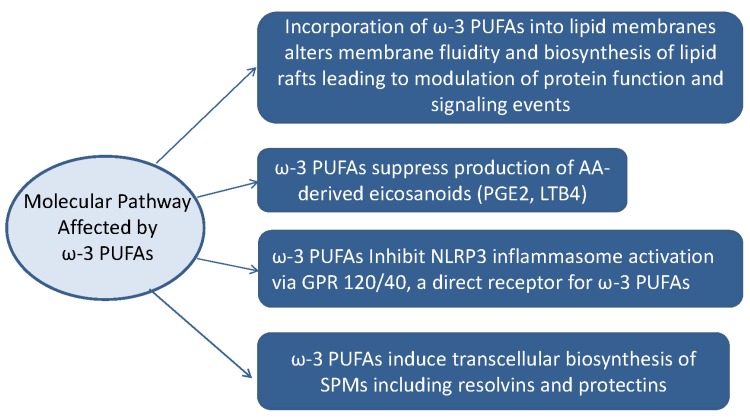
Molecular pathways affected by ω-3 PUFAs. AA: arachidonic acid; PGE2: prostagalandin E2; LTB4: leukotriene B4; GPR: G protein-coupled receptor; NLRP3: NACHT, LRR and PYD domains-containing protein 3; SPM: specialized pro-resolving mediator.

**Figure 2:**
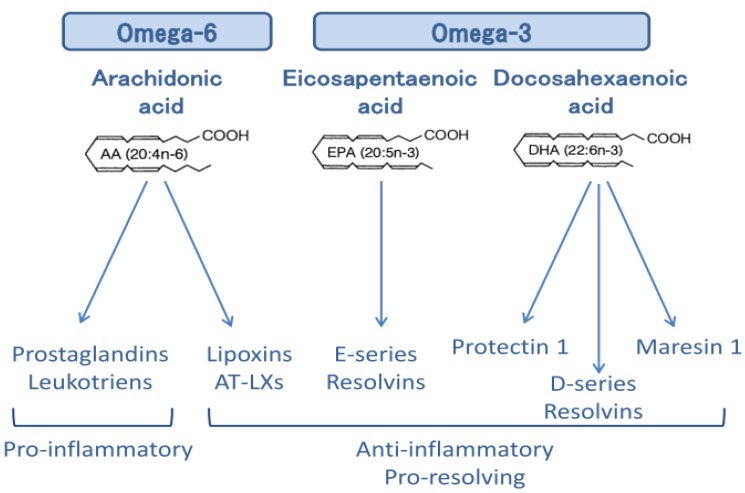
Specialized pro-resolving mediators (SPMs) derived from ω-3 and ω-6 PUFAs. AT-LXs: Aspirin-Triggered Lipoxins.

Several reports regarding the anti-inflammatory action of ω-3 PUFAs exist as summarized in Figure 1. Reduction in membrane arachidonic acid level, suppression of inflammatory eicosanoids, inflammation convergence by EPA and DHA metabolites, and suppression of cyclooxygenase (COX)-2 and 5-lipoxygenase (LOX) occur as parts of the eicosanoid-dependent anti-inflammatory mechanism. An eicosanoid-independent mechanism was recently reported [23] which is mediated through G protein-coupled receptor (GPR) 120/40 and scaffold protein β-arrestin, which are direct receptors of ω-3 unsaturated fatty acids [24]. This new mechanism inhibits IL-1β release by suppression of the NACHT, LRR and PYD domains-containing protein 3 (NLRP3) inflammasome activation [25]. In fact, NLRP3 inflammasome is reported as a novel target for docosahexaenoic acid metabolites to abrogate glomerular injury [26]. In the eicosanoid-dependent mechanism, arachidonic acid (AA)—an inflammation-induced omega-6 PUFA—is converted mainly to inflammatory mediators such as prostaglandin (PG) and leukotriene (LT) by COX. This is in contrast to the observation that lipid mediators control inflammation by converting lipids to lipoxins [26] such as lipoxin A4 (LXA4) via LOX. In recent years, research has focused on the development of lipid mediators with anti-inflammatory mechanism, such as ω-3 PUFAs, and EPA and DHA-derived molecules with inflammatory convergence functions [27]. Inflammatory responses can be self-limiting by triggering the production of a superfamily of chemical mediators called specialized pro-resolving mediators (SPMs), which include endogenous mediators of the *n*-3-derived families such as resolvins [28], protectins [29], and maresins [30], as well as arachidonic acid-derived (*n*-6) lipoxins [26], which promote the resolution of inflammation (Figure 2). Aspirin and statin positively affect these degradation pathways by generating epimeric forms of specific SPMs [31].

## 5. Combination Therapy of Aspirin and EPA

We have previously reported clinical observations regarding the treatment of three patients with IgAN with highly purified EPA and aspirin in the absence of immunosuppressants [32]. The combination therapy was effective within 2–3 months of its initiation. The success of the combined therapy may be due to the specific pharmacological action of aspirin in enhancing the production of EPA-derived anti-inflammatory lipid mediators. In addition, we previously reported successful treatment cases of anti-neutrophil cytoplasmic-associated glomerulonephritis [33] and vasculitis [34] using the combination of aspirin and EPA. Aspirin is generally used as an anti-inflammatory substance, and low-dose aspirin is currently recommended as a cardiovascular prophylactic drug. The beneficial effects of aspirin in the cardiovascular system have been attributed to its ability to block prostaglandin and prothrombotic thromboxane A2 (TXA2) generation by the acetylation of COX-1 [35]. In addition, aspirin acetylation of COX-2 alters active sites of COX-2 and permits conversion of AA to 15R-hydroxyeicosatetraenoic acid (HETE) in endothelial cells, which can be transformed to epimeric lipoxins by leukocytes. On the other hand, ω-3 PUFAs such as EPA and DHA are substrates of acetylated COX-2, which generates biosynthetic precursors to AT-resolvins [36]. The general inflammation and the inflammation convergence effect derived from the production of lipid mediators of aspirin-induced essential fatty acids might also be responsible for its efficacy as a cardiovascular prophylactic drug [37,38]. However, the mechanism of this synergistic action is not sufficiently elucidated. The synergistic action of aspirin and fish oil is possibly due to the increased production of inflammatory convergent mediators such as resolvin, protectin, and their aspirin-induced counterparts derived from EPA and DHA. Acetylation of COX-2 by aspirin promotes the production of 17R-hydroxydocosahexaenoic acid (HDHA) from 15R-hydroxyeicosatetraenoic acid (HETE), and DHA from arachidonic acid, whereas it suppresses prostaglandin production [39]. In addition, it induces the production of anti-inflammatory aspirin-induced lipoxin, 17R-HDHA, and D-series resolvin. Thus, the endogenous production and metabolism of EPA- and DHA-derived inflammatory convergent mediators may differ in the absence of aspirin [40].

## 6. Conclusions

Several recent clinical trials have provided convincing results about the effectiveness of ω-3 PUFAs in the treatment of human IgAN, whereas test results showed that the desired efficacy was not observed. The clinical application of EPA in treatment of IgAN is stagnant at present. ω-3 PUFAs such as EPA have various effects, including cardioprotective and anti-arteriosclerotic effects. In fact, the results of the JELIS trial [19] showed a further improvement in the incidence of cardiovascular events by the addition of EPA to statins for treating patients with hyperlipidemia. The incidence of cardiovascular events—a prognostic factor—is significantly higher in patients with CKD. However, the effect of EPA on pathogenesis of the primary disease and CKD is systemic. Considering its efficacy, the effectiveness of EPA in the early stage of IgA nephropathy should be investigated, and it should be actively introduced in the clinic. Lipid mediators derived from omega-3 PUFAs can be used as SPMs for the treatment of rheumatoid arthritis. We hope that the concept of SPM finds application in the treatment of renal disorders such as IgAN, and the controversy regarding the effect of EPA on the prognosis of IgAN is resolved. We believe that successful administration of EPA/aspirin combination therapy for treating IgA nephropathy might be a promising alternative to steroid replacement therapy. Therefore, well-designed molecular experiments and clinical trials should be implemented to understand the mechanisms via which ω-3 PUFAs affect this disease.

## Figures and Tables

**Table 1:** Clinical trials evaluating ω-3 polyunsaturated fatty acids (PUFAs) in patients with IgA nephropathy (IgAN). DHA: docosahexaenoic acid; EPA: eicosapentaenoic acid; GFR: glomerular filtration rate; RASB: Renin–Angiotensin–Aldosterone System Blocker.

First Author, Reference	Year	Intervention	Population	Duration of Follow-Up, Years	Main Findings
Hamazaki [11]	1984	EPA (1.6 g) + DHA (1.0 g)	10	1	EPA might be a safe and useful agent to stop the progression of IgAN
		Control: EPA/DHA (−)	10		
Alexopoulos [12]	2004	EPA (0.9 g) + DHA (0.6 g)	14	4	A “very low dose” of omega-3 PUFAs is effective in slowing renal progression in high-risk patients with IgAN
		Control: EPA/DHA (−)	14		
Sulikowsa [13]	2004	EPA (0.8 g) + DHA (0.5 g)	20	1	Omega-3 PUFAs supplementation is associated with the improvement of both renal vascular function and tubule function
Donaldio [14,15]	1994	EPA (1.9 g) + DHA (1.5 g)	55	6.4	Early and prolonged treatment with fish oil slows renal progression for high-risk patients with IgA nephropathy
	1999	Control: placebo	15	6.8	
Donaldio [16]	2001	High dose EPA (3.8 g) + DHA (2.9 g) Low dose EPA (1.9 g) + DHA (1.5 g)	36	2	Low-dose and high-dose omega-3 fatty acids were similar in slowing the rate of renal function loss in high-risk patients with IgAN
			37		
Ferraro [17]	2009	EPA + DHA (2.6 g)+ RASB	15	0.5	Omega-3 PUFAs associated with RASB reduced proteinuria in patients with IgAN more than RASB alone
		Control: RASB	15		
Moriyama [18]	2013	RASB + EPA (0.9–1.8 g)	18	1	EPA accelerates the effects of RASB and thus decreases the proteinuria observed in patients with IgAN
		Control:RASB + dilazep	20		
Bennett [19]	1989	EPA (10 g)	17	2	EPA does not alter the course of established mesangial IgA nephropathy
		Control: no treatment	20		
Pettersson [20]	1994	EPA (3.3 g) + DHA (1.8 g)	15	0.5	By 0.5 year, omega-3 PUFAs supplements resulted in a slight but significant reduction in GFR
		Control: EPA/DHA (−)	17		
Hogg [21]	2006	EPA (1.9 g) + DHA (1.5 g)	32	2	The effect of omega-3 PUFAs on proteinuria in patients with IgAN is dosage-dependent
		placebo	31		
